# ^1^H, ^13^C, and ^15^N backbone chemical shift assignments of m^7^GTP cap-bound *Leishmania* initiation factor 4E-1

**DOI:** 10.1007/s12104-020-09958-3

**Published:** 2020-06-09

**Authors:** Anissa Belfetmi, Mélissa Léger-Abraham

**Affiliations:** Department of Microbiology, Blavatnik Institute, Harvard Medical School, Boston, MA 02115 USA

**Keywords:** *Leishmania*, Translation initiation, LeishIF4E-1, NMR

## Abstract

Most of the translational control of gene expression in higher eukaryotes occurs during the initiation step of protein synthesis. While this process is well characterized in mammalian cells, it is less defined in parasites, including the ones that cause human Leishmaniasis. The *Leishmania* cap-binding isoform 1 (LeishIF4E-1) is the only isoform that binds the specific trypanosomatids-specific hypermethylated 5′ cap, called cap-4, in the human stage of the parasite life cycle. We report here the extensive NMR resonance assignment of LeishIF4E-1 bound to a cap analog, m^7^GTP. The chemical shift data constitute a prerequisite to understanding specific translation initiation mechanisms used in *Leishmania* parasites and to developing antiparasitic drugs targeting their translation initiation factors.

## Biological context

The translation initiation heterotrimeric complex, eIF4F, recruits the 5′ end of mRNAs to the small ribosomal subunit (Shirokikh and Preiss 2018). In human cells, this complex comprises a cap-binding protein, eIF4E, which specifically binds a methylated cap structure (m^7^GTP) at the 5′ end of most cellular mRNAs. A scaffold protein, eIF4G, binds eIF4E, the DEAD-box RNA helicase eIF4A, the poly-A binding protein PABP, and the multisubunit eIF3, which binds to the small ribosomal subunit. eIF4G interacts with eIF4E through a consensus motif, Y(X)_4_LΦ (where X is any amino acid and Φ is a hydrophobic residue). Despite our increasing knowledge of translation mechanisms in human cells, how the equivalent process is carried out in trypanosomatids, including *Leishmania major*, is not as well understood.

The specific factors involved in translation initiation vary depending on the developmental stage of *Leishmania* parasites. These parasites encode six highly diverged cap-binding protein isoforms (LeishIF4E-1 through −6) that significantly differ among themselves and from their orthologs in mammalian cells (Yoffe et al. 2004, 2006, 2009; Zinoviev et al. 2011). Trypanosomatids also contain a specific hypermethylated cap structure at the 5′ end of their mRNAs, called cap-4 (Reolon et al. 2019; Leiter et al. 2020). In the human developmental stage of the parasite’s life cycle, LeishIF4E-1 is the only isoform that is highly expressed and maintains cap-binding activity, suggesting that it is the functional cap-binding protein in the human infective stage (Zinoviev et al. 2011). LeishIF4E-1 does not, however, interact with any of the predicted LeishIF4G scaffold proteins, suggesting that LeishIF4E-1 is recruited to the LeishIF4F translation initiation complex through novel interactions, or that translation initiation in amastigotes proceeds through a cap-independent mechanism. A crystal structure of LeishIF4E-1 bound to a fragment of a novel IF4E interacting protein (Meleppattu et al. 2018), Leish4E-IP1, showed that the core of LeishIF4E-1 is structurally conserved when compared to eIF4E. Structural analysis, coupled with biochemical assays, revealed that Leish4E-IP1 represses cap-binding. As the cap was absent in the crystal structure, the structure did not allow us to visualize how LeishIF4E-1 interacts with the 5’ mRNA cap. Here, we report the extensive NMR resonance assignment of LeishIF4E-1 bound to a m^7^GTP cap analog.

## Methods and experiments

### Constructs

A plasmid that encodes full-length *Leishmania* IF4E-1 (LeishIF4E-1, accession number: LmjF27.1620, which is 214 amino acids in length, with a molecular weight of 24.2 kDa) that is expressed as a fusion protein with a hexahistidine (His_6_) tag, and a Tobacco Etch Virus (TEV) protease cleavage site at its N terminus, pHis_6_-TEV-LeishIF4E-1, has been previously described (Meleppattu et al. 2018). From this plasmid, we used site-specific mutagenesis to generate several tryptophan mutants by polymerase chain reaction (LeishIF4E-1 numbering: W22G, W25G, W37A, W83A, W95G and W133G).

### Sample preparation

We grew *E. coli* strain BL21 (DE3) cells transformed with this plasmid in M9 minimal media containing 95% ^2^H_2_O supplemented with ^15^NH_4_Cl (1 g/L) and ^13^C_6_-D-glucose (2 g/L), when required, as the sole nitrogen and carbon sources, respectively. We used 1 mM of isopropyl β-D-1-thiogalactopyranoside (IPTG) to induce protein expression and grew cells at 20 °C for 16 hours. We harvested and re-suspended cells in buffer containing 50 mM NaH_2_PO_4_/Na_2_HPO_4_, pH 7.8, 500 mM NaCl, 10 mM imidazole, 5 mM 2-β-mercaptoethanol, benzonase and EDTA-free protease inhibitor cocktail tablet (Roche). We lysed cells by sonication at 4 °C and centrifuged the resulting lysate. We purified the His_6_-TEV-LeishIF4E-1 protein on a nickel-affinity column (Ni-NTA; Qiagen) as previously described (Meleppattu et al. 2018). We digested the eluted protein with TEV protease to cleave the His_6_ tag for 12 h (overnight reaction). We concentrated the protein by ultrafiltration using a 10 kDa-cutoff filter (Millipore Sigma). We further purified LeishIF4E-1 from the cleaved tag using a Superdex 75 HiLoad 16/60 preparative size exclusion column (GE Healthcare Life Sciences). After size exclusion purification, we concentrated the protein, added 5 mM of m^7^GTP (Millipore Sigma) and incubated overnight. We buffer exchanged samples into an NMR buffer (50 mM NaH_2_PO_4_/Na_2_HPO_4_, pH 6.5, 100 mM NaCl, 2 mM DTT, 5 mM m^7^GTP 5% D_2_O, concentrated to 0.1–0.4 mM) and removed excess of m^7^GTP using a desalting PD-10 column (GE Healthcare). NMR spectroscopy experiments were recorded at 298 K either on Bruker or Varian spectrometers, operating at high field strengths of 600, 750, and 900 MHz, all equipped with a cryogenically cooled probe.

### NMR experiments

**Fig. 1:**
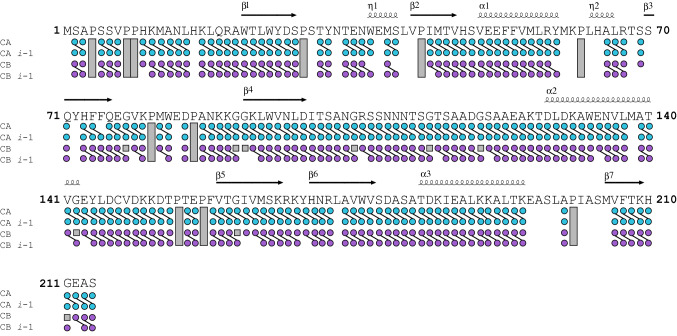
Summary of the data establishing the sequence-specific resonance assignments of LeishIF4E-1 bound to m^7^GTP. The LeishIF4E-1 secondary structure information, based on the crystal structure of the LeishIF4E-1/Leish4E-IP1 complex (PDB: 5WB5), is indicated above the sequence (α: alpha helices, η: 3_10_-helix, β: beta-strands). UniProt accession no.: E9ADE1. The resonance peaks observed in the 3D TROSY-HNCA or the 3D TROSY-HNCACB (CA:C^α^, CA *i*-1: sequential C^α^, CB: C^β^, CB *i*-1: sequential C^β^) are indicated by a dot below the amino acid sequence of LeishIF4E-1. CA and CA *i*-1 are shown in cyan, while CB and CB *i*-1 are in purple. An oblique line describes established connectivity. Grey boxes indicate unobservable signals (prolines and glycine C^β^)

**Fig. 2:**
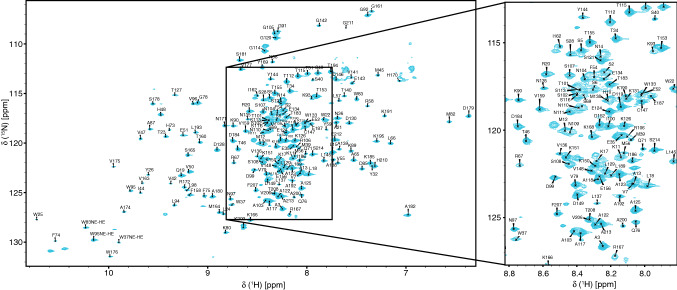
2D ^1^H–^15^N TROSY-HSQC of LeishIF4E-1 bound to m^7^GTP (25 °C, pH 6.5, 900 MHz). The amide groups of LeishIF4E-1 residues that have been assigned are indicated. The boxed area shown in the right portion of the figure corresponds to the enlargement of the low dispersion region (~8 ppm in the ^1^H dimension)

**Fig. 3:**
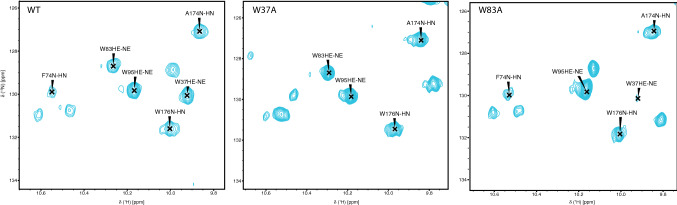
2D ^1^H–^15^N TROSY-HSQC tryptophan side chain region of LeishIF4E-1 bound to m^7^GTP. Alanine mutations of specific tryptophan residues caused chemical shift perturbations of several peaks within the vicinity of the tryptophan sidechains, with the most severe effect causing the specific tryptophan resonance to disappear. Wild-type (WT) LeishIF4E-1, LeishIF4E-1/W37A and LeishIF4E-1/W83A spectra are shown, respectively, in the left, middle and right panels

We recorded all NMR spectra at 298 K on a Varian Inova 900 MHz spectrometer or a Bruker 750 MHz spectrometer, both equipped with cryogenic probes. We processed the data using NMRPipe (Delaglio et al. 1995), and analyzed the data with CARA (Keller 2004) and SPARKY (Goddard et al. 2005). We assigned the backbone chemical shifts of ^15^N-^13^C-LeishIF4E-1 using TROSY versions of the traditional triple-resonance experiments: HNCA, HNCOCA, HNCO, HNCACO, HNCACB, and HNCOCACB (Figs. 1 and 2). We used selective amino acid labelling to identify and confirm overlapping resonances (^15^N-Xxx, where Xxx = Ala, Gly, His, Leu, Lys, Thr). We assigned three tryptophan aromatic side chains using NMR spectra collected on point mutants W37A, W83A and W95A (Fig. 3). W37 and W83 are predicted to interact with the m^7^GTP based on analysis of sequence alignments and crystal structure of cap-free or cap-bound IF4Es (Marcotrigiano et al. 1997; Volpon et al. 2006; Peter et al. 2015; Sekiyama et al. 2015; Meleppattu et al. 2018). The purification yield for the W22A, W25A and W133A mutants were not sufficient to carry out NMR experiments. We deuterated all NMR observable samples. We also used Non Uniform Sampling (NUS) in the two indirect dimensions to collect triple resonance data and used Poisson Gap Sampling to sample 12–15% of the indirect grid (Hyberts et al. 2010). We used the hmsIST program to reconstruct and process the data (Hyberts et al. 2012).

### Assignments and data deposition

We obtained the nearly complete backbone NMR assignment of LeishIF4E-1 bound to m^7^GTP, for 177 residues of the 202 non-proline residues, as shown in the ^15^N TROSY-HSQC spectrum. All chemical shifts were deposited in the BioMagResBank (www.bmrb.wisc.edu) under accession number 50250. This work paves the way to understanding how LeishIF4E-1 binds the trypanosomatid cap-4, now that two studies have reported its efficient chemical synthesis (Reolon et al. 2019; Leiter et al. 2020).
